# TAP Block Prior to Open Ventral Hernia Repair Improves Surgical Outcome

**DOI:** 10.1007/s00268-022-06508-x

**Published:** 2022-03-29

**Authors:** Leo Licari, Simona Viola, Giuseppe Salamone

**Affiliations:** 1 Department of Surgical, Oncological and Oral Sciences (DICHIRONS), Policlinico P. Giaccone, University of Palermo, Via Liborio Giuffré 5, 90127 Palermo, Italy; 2 Department of Biological, Chemical and Pharmaceutical Sciences and Technologies, University of Palermo, Palermo, Italy

## Abstract

**Background:**

Ventral hernias commonly affect patients after major abdominal surgery. To reduce postoperative pain, the effects of the transversus abdominis plane (TAP) block, epidural analgesia and medication-only protocol have been investigated. The primary outcome was the cumulative dosage of opioids (morphine milligram equivalents MME), of acetaminophen and diclofenac for postoperative pain control on postoperative day (POD) 0, 1, and 2. Secondary outcomes were length of stay (LOS) and the pain scale rating using the numeric rating scale (NRS) on POD 0, 1, and 2.

**Methods:**

The data were retrospectively extracted from the charts of the patients admitted for a surgical operation for OVHR from January 2015 to December 2019.

**Results:**

Patients receiving medication-only analgesia had longer LOS (mean 6.1 days; *p* < 0.00001). Cumulative opioid consumption was significantly lower at 24 and 48 h after surgery in the TAP block group than in the other groups (mean MME 1.9 mg and 0.7 mg, respectively; *p* < 0.05). The cumulative consumption of diclofenac was significantly lower in the TAP block group than in the others (44.1 mg; *p* ≤ 0.00001 on POD 1; 4.4 mg; *p* = 0.03 on POD 2). TAP block is more effective in pain control in POD 0 (mean NRS 5.4; *p* < 0.00001), POD 1 (mean NRS 6.1; *p* = 0.006), and POD 2 (mean NRS 4.9; *p* = 0.001) if it is performed after adopting the retromuscular technique.

**Conclusions:**

The comparison between the medication-only technique, epidural, and TAP block demonstrated the superiority of the last one for the aims considered in this study.

## Introduction

Ventral hernias commonly affect patients after major abdominal surgery. Its estimated incidence is approximately 20%, increasing to 40% in high-risk populations [1]. Perioperative pain control is one of the goals of postoperative management. Patients frequently suffer from acute and chronic pain, and this significantly affects their postoperative quality of life. Opioid analgesics were used widely until the mounting opioid crisis required physicians to provide alternative analgesia techniques [2]. Moreover, these techniques can be considered not only alternatives but also instruments to reduce the overall opioid dosage prescribed during hospitalization and after discharge.

Today, pain control research and methodologies have been enriched not only by different medications but also by different analgesic techniques. Given the heterogeneity of the techniques proposed, their absolute superiority or a precise indication for one of these techniques has not been proven for a specific surgical technique adopted to perform abdominal wall hernia repair.

To reduce postoperative pain, the effects of the transversus abdominis plane (TAP) block have been investigated. Although recent publications have mainly focused on its use in inguinal hernia repair, evidence in the literature regarding the effectiveness of the TAP block in open ventral hernia repair (OVHR) remains inadequate. This study aims to investigate and compare the outcomes of different postoperative analgesia techniques to determine one technique’s superiority in different scenarios.

## Materials and methods

The primary outcome evaluated was the cumulative dosage of opioids, in terms of morphine milligram equivalents (MME) per day, and of acetaminophen and diclofenac for postoperative pain control on postoperative day (POD) 0, 1, and 2 distinguished for specific analgesia protocol classes. Specifically, the classes assessed were medication only, epidural, and TAP block. Secondary outcomes evaluated were length of stay (LOS) for each analgesia protocol class and the pain scale rating using the numeric rating scale (NRS) on POD 0, 1, and 2 for each analgesia protocol class distinguished by surgical procedure (intraperitoneal onlay mesh (IPOM) vs. retromuscular technique).

The data were retrospectively extracted from the charts of the patients admitted for a surgical operation for OVHR from January 2015 to December 2019 in the Surgical Unit of the Policlinico Paolo Giaccone of Palermo—University of Palermo. The patients selected for the purpose met the following criteria: (1) elective surgery; (2) major diameter defect measured during the operation to be ≥5 cm; (3) surgical technique was open IPOM or open retromuscular mesh placement; and (4) postoperative analgesia protocol adopted included medication only, epidural, and TAP block. A total of 450 patients were identified in the database, and 250 met the inclusion criteria.

Description of the analgesia protocols: the medication-only protocol involved the postoperative intravenous administration of acetaminophen 1 g tris in die (tid) and diclofenac 75 mg bis in die (bid) for 24 h after surgical operation.

The epidural protocol involved its placement in the preoperative area. Infusions with 0.125% bupivacaine were initiated shortly before emergence from anesthesia and continued at 8–12 mL/h. Epidurals were discontinued variably within 24–72 h.

The TAP block was placed in the operative theater with a ultrasound-assisted technique prior to the surgical procedure. The block solution consisted of 0.375% levobupivacaine in 20 mL for each side.

Meperidine, diclofenac, and acetaminophen were used for additional pain control as needed.

Continuous variables are reported as mean ± standard deviation (SD) or median (interquartile range), and differences between the groups were tested using Student's *t *test, analysis of variance (ANOVA), or Mann–Whitney tests. Student's *t *test was used when two independent groups were compared, while ANOVA extended the findings of the *t* test to more than two groups. When ANOVA was performed, the post-hoc Tukey procedure was used to facilitate pairwise comparisons within ANOVA data, allowing us to determine between which various pairs of means showed a significant difference. Discrete variables are reported as *n* (%) and were tested using the Chi-squared test. Values of *p* less than 0.05 were considered statistically significant. Opioid dosing was calculated as morphine milligram equivalents (MME) using the Opioid Equianalgesic Calculator. All analyses were carried out using R statistical software (R Foundation for Statistical Computing, version 3.4.3, Vienna Austria).

## Results

A total of 450 patients underwent OVHR with either IPOM or retromuscular mesh placement during the study period. Of these, 250 patients met the inclusion criteria listed above in the Methods section. The postoperative analgesia protocol differed between patients. Of these, 60 (24%) patients underwent medication-only analgesia, 122 (48.8%) patients underwent epidural analgesia, and 68 (27.2%) had a TAP block. Patient body mass index (BMI), comorbidities, demographic details, and American Society of Anesthesiologists (ASA) score stratification were similar between groups (Table 1). Hernia size in the epidural group was significantly larger (mean 138.98 cm^2^ vs. 112.3 cm^2^ in the medication-only group and 128.6 cm^2^ in the TAP block group; *p* = 0.01). Patients receiving medication-only analgesia had longer in-hospital length of stay (LOS) (mean 6.1 days vs. 4.9 days in the epidural group and 3.2 days in the TAP block group; *p* < 0.00001). Patients receiving epidural analgesia had longer operative times (mean 92.3; *p* = 0.04; Table 1). The surgical techniques were similar, with the majority of patients undergoing retromuscular mesh placement (59.2%; Table 2).

**Table 1:** Demographic data

	Tot	Acetaminophen + FANS	Epidural	TAP block	*p*
*n* (%)	250	60 (24)	122 (48.8)	68 (27.2)	
Age, y, mean, ± SD	63.3 ± 12.3	66.5 ± 13.2	61.7 ± 10.1	62.8 ± 11.7	n.s
Bmi, kg/m2, median	27	28	25	22	n.s
Sex, *n* (%)					n.s
*m*	122 (48.8)	28 (46.6)	55 (45.1)	39 (57.3)	
*f*	128 (51.2)	32 (53.4)	67 (54.9)	29 (42.7)	
Comorbidities, n (%)					n.s
Hypertension	105 (42)	27 (45)	51 (41.8)	27 (39.7)	
Diabetes	95 (38)	22 (36.7)	47 (38.5)	26 (38.2)	
Cardio-vascular diseases	80 (32)	28 (46.6)	32 (26.2)	20 (29.4)	
Copd	30 (12)	5 (8.3)	18 (14.7)	7 (10.3)	
Smokers, *n* (%)	68 (27.2)	20 (33.3)	31 (25.4)	17 (25%)	n.s
Hernia size (cm2), mean ± SD	126.6 ± 2.8	112.3 ± 3.7	138.8 ± 2.6	128.6 ± 2.2	0.01
LOS mean ± SD	4.7 ± 1.4	6.1 ± 1.7	4.9 ± 1.1	3.2 ± 0.9	<0.00001
operation time (min) mean ± SD	90.1 ± 1.7	88.9 ± 8.4	92.3 ± 8.4	90.1 ± 10.5	0.04
ASA, *n* (%)					
I	–	–	–	–	–
II	63 (25.2)	12 (20)	31 (25.4)	20 (29.4)	n.s
III	175 (70)	42 (70)	71 (58.2)	62 (91.2)	n.s
IV	12 (4.8)	1 (1.6)	7 (5.7)	4 (5.9)	n.s

**Table 2:** Pain rating using numeric rating scale (NRS)

	IPOM	Retromuscular	*p*
Acetaminophen + FANS n (%)	13 (5.2)	47 (18.8)	
Epidural *n* (%)	39 (15.6)	83 (33.2)	
TAP block *n* (%)	50 (20)	18 (7.2)	
Total *n* (%)	102 (40.8)	148 (59.2)	
NRS POD 0			
Acetaminophen + FANS mean ± SD	7.2 ± 1.0	7.9 ± 1.1	0.03#
Epidural mean ± SD	6.9 ± 0.9	7.6 ± 1.1	0.0001#
TAP block mean ± SD	7.6 ± 1.0	5.4 ± 0.5	<0.00001#
*p*	0.001*	<0.00001*	
NRS POD 1			
Acetaminophen + FANS mean ± SD	6.9 ± 0.9	7.1 ± 0.9	0.29#
Epidural mean ± SD	6.8 ± 0.9	7.1 ± 1.4	0.13#
TAP block mean ± SD	6.2 ± 1.1	6.1 ± 0.8	0.26#
*p*	0.2*	0.006*	
NRS POD 2			
Acetaminophen + FANS mean ± SD	5.7 ± 1.0	5.8 ± 0.4	0.26#
Epidural mean ± SD	5.8 ± 0.9	5.8 ± 1.3	0.5#
TAP block mean ± SD	5.1 ± 0.9	4.9 ± 0.7	0.21#
*p*	0.002*	0.001*	

*ANOVA

^#^t-Student

On POD 0, the mean cumulative dose used for opioids between groups was similar (*p* = 0.28). In contrast, the mean cumulative dose for diclofenac and acetaminophen was higher in the medication-only group (Table 3).

**Table 3:** Cumulative dose of opioids, diclofenac and acetaminophen

	Acetaminophen + FANS	Epidural	TAP block	*p*
POD 0				
Opioid *n* (%)	40 (66.7)	71 (58)	42 (61.2)	
Cumulative dose MME, mg, mean ± SD	9.2 ± 7.6	7.5 ± 7.3	8.8 ± 6.8	0.28*
Diclofenac *n* (%)	60 (100)	15 (12.3)	6 (8.8)	
Cumulative dose, mg, mean ± SD	150	15.4 ± 43.1	6.6 ± 21.4	<0.00001*
Acetaminophen *n* (%)	60 (100)	10 (8.2)	12 (17.6)	
Cumulative dose, mg, mean ± SD	3000	172.1 ± 612.4	397.1 ± 916.5	<0.00001*
POD 1				
Opioid *n* (%)	52 (86.6)	31 (25.4)	13 (19.1)	
Cumulative dose MME, mg, mean ± SD	11.6 ± 6.4	4.8 ± 8.4	1.9 ± 3.9	<0.00001*
	11.6 ± 6.4	4.8 ± 8.4		<0.00001§
	11.6 ± 6.4		1.9 ± 3.9	<0.00001§
		4.8 ± 8.4	1.9 ± 3.9	0.02§
Diclofenac *n* (%)	47 (78.3)	51 (41.8)	20 (29.4)	
Cumulative dose, mg, mean ± SD	117.5 ± 62.3	62.7 ± 74.3	44.1 ± 68.8	<0.00001*
	117.5 ± 62.3	62.7 ± 74.3		0.00001§
	117.5 ± 62.3		44.1 ± 68.8	<0.00001§
		62.7 ± 74.3	44.1 ± 68.8	0.53§
Acetaminophen *n* (%)	35 (58.3)	48 (39.3)	25 (36.8)	
Cumulative dose, mg, mean ± SD	1750 ± 1491.5	1180.3 ± 1471.6	1102.9 ± 1457.2	0.16*
POD 2 medication				
Opioid *n* (%)	25 (41.6)	19 (15.5)	7 (10.3)	
Cumulative dose MME, mg, mean ± SD	5.3 ± 7.0	3.1 ± 7.3	0.7 ± 2.1	0.000169*
	5.3 ± 7.0	3.1 ± 7.3		n.s.§
	5.3 ± 7.0		0.7 ± 2.1	0.00002§
		3.1 ± 7.3	0.7 ± 2.1	0.04§
Diclofenac *n* (%)	5 (8.3)	21 (17.2)	4 (5.8)	
Cumulative dose, mg, mean ± SD	8.8 ± 31.2	19.1 ± 44.8	4.4 ± 17.8	0.02*
	8.8 ± 31.2	19.1 ± 44.8		0.18.§
	8.8 ± 31.2		4.4 ± 17.8	0.43.§
		19.1 ± 44.8	4.4 ± 17.8	0.03§
Acetaminophen *n* (%)	12 (20)	17 (13.9)	3 (4.4)	
Cumulative dose, mg, mean ± SD	250 ± 540.7	229.5 ± 640.1	88.2 ± 448.4	0.18*

*ANOVA

^#^*t*-Student

^§^Test Siegel-Tukey

The comparison of the means of the numeric rating scale (NRS) for each analgesia technique between the surgical groups showed that the mean was significantly lower in the IPOM group treated with the medication-only protocol and epidural (7.2 vs. 7.9; *p* = 0.03; 6.9 vs. 7.6; *p* = 0.0001). In contrast, it was lower in the retromuscular group treated with the TAP block technique (5.4 vs. 7.6; *p* < 0.00001). ANOVA of the analgesia techniques performed in both the IPOM and retromuscular groups showed a significant difference with *p* = 0.001 and *p* < 0.00001, respectively. The post hoc analysis with the Siegel–Tukey test showed that, in the IPOM group, the mean NRS was significantly lower in patients treated with the epidural technique than TAP block (6.9 vs. 7.6; *p* = 0.01). In contrast, in the retromuscular group, the mean NRS detected when a TAP block was performed was significantly lower than epidural (5.4 vs. 7.6; *p* < 0.00001) and the medication-only protocol (5.4 vs. 7.9; *p* < 0.00001; Table 2).

On POD 1, the mean cumulative dose for opioid use was significantly lower in the TAP block and epidural groups (mean MME 1.9 and 4.8 mg, respectively) than in the medication-only group (mean MME 11.6 mg; *p* < 0.00001). Additionally, the difference between the TAP block and epidural groups was revealed to be significant (*p* = 0.02). Similarly, the mean cumulative dose of diclofenac used was lower in the TAP block and epidural groups than in the medication-only group (44.1, 62.7, and 117.5 mg, respectively; *p* ≤ 0.00001). No differences were found between the TAP block and the epidural group when compared. Similar conclusions were found regarding the mean acetaminophen cumulative dose of 1102.9 mg in the TAP block group, 1180.3 mg in the epidural group, and 1750 mg in the medication-only group (*p* = 0.16; Table 3).

No significant differences were found following a comparison of the means of NRS for each analgesia technique between surgical groups, but in the retromuscular group, the comparison of the means of the different analgesia techniques tested with ANOVA returned a significant *p *value (*p* = 0.006). The post hoc Siegel–Tukey test showed significantly lower NRS from adopting the TAP block technique vs. the epidural and medication-only protocol (6.1 vs. 7.1; *p* = 0.003 and 6.1 vs. 7.1; *p* = 0.002; Table 2).

On POD 2, the mean cumulative dose of opioid usage was significantly lower in the TAP block group than in the epidural group (0.7 vs. 3.1 mg; *p* = 0.04); similarly, it was lower than the medication-only group (0.7 vs. 5.3 mg; *p* = 0.00002). The mean cumulative dose of diclofenac was lower in the TAP block group than in the epidural group (4.4 vs. 19.1 mg; *p* = 0.03). No differences were observed between groups with regard to the mean cumulative dose of acetaminophen (Table 3).

As in POD1, no significant differences were found following a comparison of the means of NRS of each analgesia technique between surgical groups. In the IPOM group, the comparison of the means for each analgesia technique tested with ANOVA returned a significant p-value (*p* = 0.002). The post hoc Siegel–Tukey test showed significantly lower NRS following the adoption of the TAP block technique than the epidural protocol (5.1 vs. 5.8; *p* = 0.03). Similarly, in the retromuscular group, the comparison of the means tested with ANOVA returned a significant *p *value (*p* = 0.001). The post hoc Siegel–Tukey test showed significantly lower NRS following the adoption of the TAP block technique vs. the epidural and medication-only protocol (4.9 vs. 5.8; *p* = 0.0005 and 4.9 vs. 5.8; *p* = 0.0007; Table 2).

## Discussion

Reducing postoperative pain and opioid use severely impacts clinical outcomes. The reduction in the side effects linked to prolonged medication use can improve hospital LOS, early mobilization, and postoperative quality of life [3, 4].

The aim of this study was to determine the superiority of one postoperative analgesia technique between TAP block, epidural, and only drugs for OVHR post-surgical pain control. We demonstrated that patients who received TAP blocks had decreased LOS and decreased cumulative opioid use and pain scores compared to patients who received other analgesia techniques. These findings are particularly evident on POD 1 and 2.

We found that cumulative opioid consumption was significantly lower at 24 and 48 h after surgery in the TAP group than in the other groups. Similarly, the cumulative consumption of diclofenac was significantly lower in the TAP group than in the others. Moreover, we found that the TAP block was more effective in pain control in POD 0, 1, and 2 if it was performed after adopting the retromuscular technique. Similarly TAP block is effective in pain control in POD 2 if it is was performed adopting open IPOM technique.

These results are similar to previous studies that showed TAP blocks in laparoscopic surgery are effective in reducing postoperative opioid consumption and pain scores. Milone et al. [5] studied TAP blocks in inguinal hernia repair and found that TAP blocks reduced early postoperative pain scores but did not change pain scores at 24 h postoperation. Fields et al. [6] found that TAP blocks lose effect completely at 24 h. Our findings differ from these previous results.

These differences can be explained by perhaps analyzing the surgical technique adopted. Our study found differences in NRS between IPOM and retromuscular technique in OVHR. Fields et al. [6] reported their results in a case series studying LVHR. Fields et al. [6] performed a randomized controlled trial (RCT) among 100 patients; 52 individuals received a visual-guided TAP block with 0.25% bupivacaine (50 mL), and the control group (48 patients) received a placebo injection. Both groups were similar in terms of biometric and perioperative data. The pain level was measured 1 and 24 h after surgery. The authors did not reveal a significant difference, but the patients in the TAP block group needed fewer opioids.

Similar results were discussed by Sinha et al. [7], Jain et al. [8], Bhatia et al. [9], and Paash et al. [10]. In contrast to Karamanos et al. [11], we found that epidural analgesia resulted in a statistically significant decrease in postoperative pain levels on POD 0 instead of 1, but only in the IPOM group. They also suggested that epidural analgesia is associated with a higher incidence of postoperative complications and an increased hospital LOS.

However, no RCTs to date have compared various postoperative OVHR analgesia techniques and their effects on efficacy or safety. This is, to our knowledge, one of the largest studies to evaluate the impact of TAP block, epidural analgesia, and medication-only analgesia in patients undergoing open abdominal wall reconstruction for incisional hernias.

The relatively small sample size and retrospective study design are this study’s limitations.

In addition, we did not collect data on clinical differences, such as postoperative nausea and vomiting. Future studies should assess the effects of TAP blocks on other clinically significant end points, such as postoperative nausea and vomiting and complication rates after epidural and TAP blocks [12, 13].

In conclusion, TAP blocks prior to OVHR reduce the cumulative opioid use and the cumulative dose of other medications administered on demand for pain control, such as diclofenac. The TAP block reduces the LOS after OVHR. The TAP block is associated with the lowest pain score detected with NRS, especially if the retromuscular surgical technique is performed. The comparison between the medication-only technique, epidural, and TAP block demonstrated the superiority of the last one for the aims considered in this study.
